# Motor cortex facilitation: a marker of attention deficit hyperactivity disorder co-occurrence in autism spectrum disorder

**DOI:** 10.1038/s41398-019-0614-3

**Published:** 2019-11-13

**Authors:** Ernest V. Pedapati, Lindsey N. Mooney, Steve W. Wu, Craig A. Erickson, John A. Sweeney, Rebecca C. Shaffer, Paul S. Horn, Logan K. Wink, Donald L. Gilbert

**Affiliations:** 1 Divisions of Child and Adolescent Psychiatry, Cincinnati Children’s Hospital Medical Center, Cincinnati, OH USA; 2 Division of Neurology, Cincinnati Children’s Hospital Medical Center, Cincinnati, OH USA; 3 Department of Psychology, University of California, Davis, CA USA; 4 Department of Psychiatry, University of Cincinnati College of Medicine, Cincinnati, OH USA; 5 Developmental and Behavioral Pediatrics, Cincinnati Children’s Hospital Medical Center, Cincinnati, OH USA; 6 Divisions of Biostatistics and Epidemiology, Cincinnati Children’s Hospital Medical Center, Cincinnati, OH USA

**Keywords:** Diagnostic markers, Physiology

## Abstract

The neural correlates distinguishing youth with Autism Spectrum Disorder (ASD-) and ASD with co-occurring Attention Deficit Hyperactivity Disorder (ASD+) are poorly understood despite significant phenotypic and prognostic differences. Paired-pulse transcranial magnetic stimulation (TMS) measures, including intracortical facilitation (ICF), short interval cortical inhibition (SICI), and cortical silent period (CSP) were measured in an age matched cohort of youth with ASD- (*n* = 20), ASD + (*n* = 29), and controls (TDC) (*n* = 24). ASD− and ASD+ groups did not differ by IQ or social functioning; however, ASD+ had significantly higher inattention and hyperactivity ratings. ICF (higher ratio indicates greater facilitation) in ASD+ (Mean 1.0, SD 0.19) was less than ASD− (Mean 1.3, SD 0.36) or TDC (Mean 1.2, SD 0.24) (F2,68 = 6.5, *p* = 0.003; post-hoc tests, ASD+ vs either TDC or ASD−, *p* ≤ 0.05). No differences were found between groups for SICI or age corrected active/resting motor threshold (AMT/RMT). Across all ASD youth (ASD− and ASD+), ICF was inversely correlated with worse inattention (Conners-3 Inattention (r = −0.41; *p* < 0.01) and ADHDRS-IV Inattention percentile (r = −0.422, *p* < 0.01) scores. ICF remains intact in ASD− but is impaired in ASD+. Lack of ICF is associated with inattention and executive function across ASD. Taken with the present findings, ADHD may have a distinct electrophysiological “signature” in ASD youth. ICF may constitute an emerging biomarker to study the physiology of ADHD in ASD, which may align with disease prognosis or treatment response.

## Introduction

Attention Deficit Hyperactivity Disorder (ADHD) is the most common co-occurring psychiatric condition diagnosed in Autism Spectrum Disorders (ASD) with an estimated 37–78% of individuals with ASD have symptoms of inattention, hyperactivity, and/or impulsivity and meet criteria for ADHD^1^. Youth with a co-diagnosis of ADHD and ASD (ASD+) have higher rates of hospitalization, medication treatment, and behavioral therapy than ASD alone (ASD−)^2^.

There remains an open question regarding the best methods to classify ADHD co-occurrence in ASD in the clinical setting^3^. Despite the robust incidence of ADHD symptomatology in ASD, studies have found that ASD+ is under recognized and less frequently treated in ASD youth compared to otherwise typical children^4^. When the diagnosis is recognized, ASD+ tend to respond poorly to conventional treatments, such as stimulants and behavioral therapies, compared to ADHD present in otherwise typically developing children (TDC)^5–7^. The diagnosis and treatment of ASD+ is limited by a poor understanding of the neurobiology underlying the phenotypic differences in symptoms, severity, and treatment response from ASD-^8^.

One approach has been to identify quantitative, brain-based biomarkers of ADHD symptoms and compare these in children with ASD+ vs ADHD to identify objective measures which might be unique to ASD vs those which might overlap. For example, increased frontocentral theta activity (and to a lesser extent decreased beta activity) as measured by quantitative electroencephalography (EEG) is robustly related to inattention in ADHD, but has not been replicated in either ASD− or ASD+^9^. Yet, since attentional problems are characteristic of both ADHD and ASD+, computerized testing demonstrates increased intrasubject variability in reaction time tasks in comparison to typical and ASD− groups^10^. Measures of social cognition, as expected, are more impaired in ASD+ than ADHD. Tye et al^11^. studied face processing through event related potentials in ASD−, ASD+, TDC, and ADHD. The group identified abnormalities in facial gaze processing and neuronal lateralization in the ASD groups (ASD+ and ASD−) compared to either ADHD or TDC. However, both ADHD affected groups (ASD+ and ADHD) groups had measured deficits in latency measures associated with early visual attention compared to ASD+ and TDC.

An alternative method to probe the underlying neural processes driving hyperactivity, inattention, and impulsivity symptoms in ASD+ is transcranial magnetic stimulation (TMS) interrogation of the motor cortex (M1) to probe cortical excitability. To date, this has uncovered various relationships with ADHD symptoms. Previous work by our laboratory using paired pulse TMS has demonstrated that reduced short interval cortical inhibition (SICI), is associated with both greater clinical symptom severity and more impaired motor development in children with ADHD^12^. The consistency of these M1 SICI findings across multiple research groups was recently confirmed by meta-analysis^13^. However, it is unknown whether this relationship between reduced M1 SICI and ADHD is also present in ASD+.

Thus, the goal of our study was to more clearly delineate neurophysiological differences in resting motor physiology, as a marker of cortical excitability, between ASD+ and ASD− to better understand what engenders phenotypic differences in hyperactivity, inattention, and other executive functioning deficits. Based on our previous work and prominent ADHD symptoms in ASD+, we expected to find decreased SICI in ASD+ compared to ASD− and control subjects. This would support the idea that there are overlapping neural processes related to hyperactivity, inattention, or impulsivity. Alternatively, widely divergent physiological findings in ASD+ from ASD− and ADHD may have implications for prognosis and treatment.

## Methods and materials

### Participants

Subjects with a known diagnosis of ASD between the ages of 8 and 21 years of age were recruited from a pediatric psychiatry clinic at a tertiary children’s medical center specializing in ASD and neuropsychological assessment. All ASD subjects had previously been diagnosed at the medical center following a standardized multidisciplinary evaluation which included the Autism Diagnostic Observation Schedule (ADOS)^14^. Exclusion criteria included severe medical or neurological illness, substance use disorder, schizophrenia, bipolar disorder, or major depression. Subjects were excluded if they were taking medications affecting glutamate neurotransmission (i.e., riluzole, memantine, acamprosate, topiramate, or amantadine). Any subject prescribed a stimulant medication was asked to hold their dose for 24 h prior to the study date.

For TDC controls, a cohort of subjects with no psychiatric or developmental diagnoses was recruited from pre-existing data from our pediatric TMS research program. Subjects were matched as closely as possible by age and sex, blinded to neurophysiological results. Control subjects have a documented full-scale IQ > 80 (for participants under age 12 years) or are enrolled in regular classrooms with no special educational needs. Absence of psychiatric or developmental disorders is confirmed by structured diagnostic interviews as well as direct interview by board certified pediatric neurologists and psychiatrists on the research team. Based on previous neurophysiology experiments including paired pulse TMS measures in TDC and ADHD^12^ and event related potential studies in ASD− and ASD+^11^, sample sizes of approximately 20 per a group would have 80% power to detect a large effect size based on a two-tailed alpha criterion of 0.05. Though we had interest including an ADHD-only cohort from our previous studies, the ages and sex distribution were unevenly matched. All TMS data were generated using the same equipment, using the same experimental techniques. The IRB of Cincinnati Children’s Hospital Medical Center approved the conduct of this study (ClinicalTrials.gov number, NCT02874690). We obtained written informed consent from all participants and their parents.

### Diagnosis and clinical measures

On the day of the study visit, the primary study physician, performed both a medical history and physical evaluation, reviewed the previous baseline testing and confirmed the diagnosis of ASD based on the Diagnostic and Statistical Manual of Mental Disorders (DSM-5) criteria^15^. In addition to ADOS testing, cognitive testing results (IQ) were obtained from previous clinical or research tests (within the past 5 years) performed at our institution or from psychological assessments from the patient’s school and represent scores from the Stanford-Binet^16^, Wechsler Intelligence Scale for Children^17^, or Wechsler Adult Intelligence Scale^18^.

On the day of the study, parents or guardians were asked to complete the Social Responsiveness Scale (SRS)^19^, the Vineland Adaptive Behavior Scales-2nd Edition (VABS-II)^20^, Conners Parent Rating Scale–Revised (CRS; ADHD severity T score accounts for age and gender)^21^, and Social Communication Questionnaire (SCQ)^22^ to assess adaptive behavior, problem behaviors and competencies, attention, health, and social behaviors. The Physical and Neurological Exam for Soft Signs (PANESS) was also used to assess subtle soft neurological signs and motor skills in children that had demonstrated aberrance in ASD and ADHD^23^.

To be co-diagnosed with ADHD (ASD+), the patient needed to have met (1) DSM-5 criteria for ADHD Combined presentation (314.02), ADHD Predominantly inattentive Presentation (314.00), or ADHD Predominantly hyperactive/inattentive presentation (314.01) and (2) have impairment in a moderate or severe level of function attributed to the ADHD symptoms as understood by clinical history and standardized measures (CPRS, ADHD-IV rating scales). In our matched TDC sample, subjects were excluded if they were suspected of ADHD (i.e., elevated CPRS or DSM-IV or five criteria), diagnosed with a developmental or other neuropsychiatric disorder, or reported ADHD diagnoses among first-degree relatives.

### Computerized attention testing

Attentional function was assessed using the KiTAP^24^. KiTAP is an easy to follow computer-assisted test battery for non-verbal attentional functioning across several key domains and has been validated in a neurodevelopmental population^25^. Subtests were performed in the following order: Alertness, Distractibility, Flexibility, and Go/No Go. Following previous methods in a lower-functioning population^25^, the shortest subtests will be administered first to give the widest number of children the greatest chance to complete an entire subtest.

### Transcranial magnetic stimulation

TMS experiments were performed using two Magstim 200 stimulators (Magstim Co., New York, NY, USA) connected through a Bistim module to a 90-mm circular coil. Circular coils produce similar qualitative patterns of inhibition and facilitation from motor cortex stimulation^26^. Electromyography (EMG) was recorded from the dominant first dorsal interosseous (FDI) muscle with surface electrodes, amplified, filtered (100/1000 Hz) (Coulbourn Instruments, Allentown, PA) and stored for analysis using Signal^®^ software and a Micro1401 interface (Cambridge Electronic Design, Cambridge, UK), as we have described previously^12^. Initially, single pulse was performed using a 90-mm circular coil (anterior posterior orientation) over the dominant primary motor cortex to elicit a motor evoked potential (MEP) and to determine active and resting motor thresholds (AMT, RMT) using standard methods^27^. A site for stimulation was marked using a wax pencil that could produce clear MEPs with the lowest possible stimulation intensity.

Subjects (primarily due to younger age and motor cortex immaturity) who necessitated greater than 100% stimulator output to measure either threshold or single/paired pulse were excluded from additional TMS measures. After establishing individual subject AMT and RMT, thirty trials of single or paired pulse stimulation at an interval of 6-7 s with one of three conditions in pseudorandom order: (1) single (test) pulse, (2) paired pulse (condition/test) at an interstimulus interval (ISI) of 3 milliseconds, and (3) paired pulse (condition/test) at an ISI of 10 milliseconds were administered. The test pulse was set at 120% of participant RMT and the conditioning pulses set at approximately 60% of RMT. Previous work in our laboratory found that conditioning pulses greater than 60% led to stronger inhibition in clinical and control youth compared to “dose” intensity curves in healthy adults^12,28,29^. Thus, as in our previous studies, we elected to use a slightly less efficient conditioning pulse that would yield a greater spread of ratios in children leading to a higher opportunity to investigate group differences of interest and maintain the ability to make comparisons with our previous datasets. SICI and ICF were expressed as a ratio of the mean peak to peak MEP amplitude produced by the 3 ms and 10 ms ISI, respectively, to the mean peak to peak MEP amplitude produced by the test pulse condition alone. Next, cortical silent period (CSP) was measured following administration of six pulses at 150% of AMT during a moderate FDI muscle contraction (~50% maximal force). Average CSP was calculated from the average of onset and offset of the silent period of each of 6 rectified EMG tracing^30^. A 16-point review of systems was used pre and post TMS to assess for any adverse effects^31,32^.

### Statistical analysis

All statistical analyses were performed with SPSS version 24 (IBM Corporation, Chicago, IL) and R version 3.6.0. Analysis code and deidentified datasets are available on request. All data were assessed for normality with the Shapiro-Wilk test. The primary outcome of this study was a comparison of TMS evoked measures (RMT, AMT, SICI, ICF, and CSP) between ASD−, ASD+, and TDC groups using a one-way ANOVA and adjusted *p*-values were calculated using Tukey post-hoc analysis. If Levene’s Test of Homogeneity of Variance was violated, then a one-way Welch ANOVA was conducted and adjusted p-values were obtained following Games-Howell post-hoc testing. Clinical measures (i.e., ADHD measures), computerized testing (i.e., KiTap), motor function (PANNES total), and demographics were compared between ASD− and ASD+ using a series of independent *t*-tests. The Holm method was implemented to generate adjusted *p*-values to correct for multiple comparisons between groups. Pearson’s correlation coefficient (r) or Spearman rank coefficient (based on normality testing) was used to determine associations between TMS measures and behavioral data and stratified by diagnosis. To control for type I errors, we implemented the false discovery rate procedure^33^ to calculate adjusted *p*-values from the correlation matrix of demographics, clinical, and TMS physiology measures.

## Results

### Demographic data

The final sample for analysis included 49 ASD subjects (20 ASD− (one female) and 29 ASD+ (one female) and 24 (one female) matched TDC subjects for TMS measures. No significant differences in age, gender, or handedness were identified between groups. Demographics and clinical measures are presented in Table 1. This final sample does not include eight younger subjects (two ASD−, six ASD+; mean age = 9.4, SD = 1.6), who were excluded from analysis due to thresholds requiring greater than 100% stimulator output to complete TMS measures. One ASD- subject could not complete the TMS session due to cooperation unwillingness. CSP for two subjects was not available due to technical difficulties while recording the data. There were no significant adverse events related to TMS administration.

**Table 1:** Demographic and clinical features of subjects by group

		ASD (*n* = 20)	ASD+ (*n* = 29)	*t* value	*P*
Age (years)	Mean ± SD	16.1 ± 3.3	16 ± 2.8	0.057	0.955
Gender^a^	Male: female	19:1	28:1		0.655
Handedness^a^	R:L	17:3	21:4		0.391
Full-scale IQ	Mean ± SD	85.8 ± 11.9	88.3 ± 24.9	−0.343	0.734
*Vineland adaptive behavior scale*					
Composite	Mean ± SD	71.7 ± 12.4	69.5 ± 10.4	0.653	0.517
Communication	Mean ± SD	71.4 ± 13.1	72.0 ± 12.3	−0.178	0.860
Daily living skills	Mean ± SD	77.3 ± 20.3	75.2 ± 13.1	0.432	0.668
Socialization	Mean ± SD	28.5 ± 6.7	27.6 ± 8.2	0.698	0.489
PANESS	Mean ± SD	38.3 ± 12.2	46.1 ± 12.5	−2.138	0.039^b^
SCQ Score	Mean ± SD	20.7 ± 6.9	21.3 ± 8.1	−0.504	0.617
SRS Score	Mean ± SD	88.9 ± 27.4	91.7 ± 27.2	−0.705	0.484
*Aberrant behavior checklist*					
Irritability	Mean ± SD	6.1 ± 7.2	8.4 ± 8.2	−1.141	0.260
Social withdrawal	Mean ± SD	13.5 ± 8.8	11.5 ± 7.2	0.681	0.499
Stereotypy	Mean ± SD	5.1 ± 4.8	5.2 ± 5.4	−0.184	0.855
Hyperactivity	Mean ± SD	6.5 ± 5.5	14.8 ± 7.7	−4.288	<0.001^c^
Inappropriate speech	Mean ± SD	3 ± 2.9	4.6 ± 3.4	−1.853	0.070
*Conners-3 parent*					
Inattention	Mean ± SD	60.5 ± 12.3	75.2 ± 10.8	−4.900	<0.001^c^
Hyper/impulsive	Mean ± SD	57.1 ± 16.3	77.0 ± 14.6	−4.957	<0.001^c^
Learning problems	Mean ± SD	65.2 ± 13.1	67.6 ± 12.4	−0.728	0.470
Executive function	Mean ± SD	53.2 ± 9.4	67.2 ± 10.4	−5.642	<0.001^c^
Defiance aggression	Mean ± SD	53.2 ± 13.4	58.7 ± 16.3	−1.346	0.185
Peer relations	Mean ± SD	82.9 ± 12.5	86.0 ± 7.1	−1.178	0.245
ADHD-IV rating scale					
Inattentive percentile	Mean ± SD	63.9 ± 28.2	89.3 ± 14.3	−5.048	<0.001^c^
Hyperactivity percentile	Mean ± SD	52.8 ± 29.4	83.8 ± 20.0	−5.517	<0.001^c^
Total percentile	Mean ± SD	61.8 ± 27.2	89.1 ± 16.3	−5.737	<0.001^c^

*ASD* Autism spectrum disorder, *ASD−* without ADHD co-occurrence, *ASD+* with ADHD co-occurrence, *PANESS* physical and neurological examination of soft signs, *SCQ* social communication questionnaire, *SRS* social responsiveness scale

^a^Fisher Exact Test; superscript indicates adjusted *p*-values following Holm correction as follows: *p* > 0.05 (non-significant following correction)

^b^*p* < 0.01

^c^*p* < 0.001

Children in the ASD− group were taking the following other medications: selective serotonin reuptake inhibitors (*n* = 8, 40%) and antipsychotics (*n* = 6, 30%) while children in the ASD+ group were taking the following medications: selective serotonin reuptake inhibitors (*n* = 17, 59%); antipsychotics (*n* = 8, 28%); stimulants (*n* = 17, 59%); and alpha-2 agonists (*n* = 7, 24%).

### Parent reported measures and computerized testing

As expected, the ASD+ group had marked inattention, hyperactivity, and executive function symptoms across all ADHD scales (see Table 1) compared to ASD−. In addition, ASD+ had worse PANESS scores indicating greater motor dysfunction. Outside of ADHD symptoms, the ASD− and ASD+ had similar IQ, adaptive function, and social scores with no significant differences between VABS, SCQ, SRS, or ABC subscores (excluding ABC-hyperactivity; see Table 1). No significant differences on KiTap computerized testing (Alertness, Distractibility, Flexibility, and Go-No Go measures) between ASD- and ASD+ were found following FDR adjustment.

### Comparison between groups: TMS measures

Comparisons of resting motor physiology data from ASD+, ASD−, and TDC subjects are shown in Table 2. Of significance, ICF ratio was statistically significant between groups (Welch’s F(2, 38.62) = 6.84, *p* = 0.003). ICF ratio was higher in the ASD- group (1.27 ± 0.36) and TDC (1.18 ± 0.24) compared to the ASD+ group (1.01 ± 0.19). Games-Howell post-hoc analysis revealed that ASD+ was reduced compared to both ASD− (−0.26, 95% CI −0.48 to −0.05, *p* = 0.016) and TDC (−0.17, 95% CI (−0.32 to −0.03)), *p* = 0.018). No significant difference between magnitude of MEP raw amplitudes (mV) were identified between ASD− and ASD+ groups for single test pulses with no conditioning pulse (t = 0.80; *p* = 0.43), 3 ms ISI conditioning pulse (t = −0.09; *p* = 0.93), and 10 ms ISI conditioning pulse (t = 1.36; *p* = 0.18).

**Table 2:** Comparison of resting motor physiology between ASD+, ASD−, and controls

Measure	ASD (*n* = 20)	ASD+ (*n* = 29)	TDC (*n* = 24)	Statistic	Post-hoc test
Age	16.1 ± 3.3	16 ± 2.8	16.3 ± 6.5	F = 0.02; *p* = 0.98	
Handedness	17:3	21:4	24:0	X = 4.3; *p* = 0.12	
RMT (%)	51 ± 10.3	48.1 ± 8.2	55.5 ± 12	F = 3.5; *p* = 0.03^a^;	ASD + vs TDC, *p* = 0.03
AMT (%)	36.2 ± 8.9	34.7 ± 6.6	39.9 ± 8.4	F = 2.9; *p* = 0.07	
SICI (%)	0.61 ± 0.31	0.63 ± 0.27	0.56 ± 0.25	F = 0.5; *p* = 0.62	
ICF (%)	1.27 ± 0.36	1.01 ± 0.19	1.18 ± 0.24	F = 6.5; *p* = 0.003^b^	ASD + vs. TDC, *p* = 0.018 ASD + vs. ASD-; *p* = 0.016
CSP (ms)	53 ± 39	65 ± 46	82 ± 34	F = 2.8; *p* = 0.069	

Results of ANOVA and post-hoc multiple comparisons (all values expressed as mean ± SD). SICI and ICF expressed as percentage of conditioned pulse amplitude versus baseline amplitude.

*ASD* autism spectrum disorder, *ASD−* without ADHD co-occurrence, *ASD* *+*, with ADHD co-occurrence, *TDC* typically developing control, *TMS* transcranial magnetic stimulation, *MSO* maximum stimulator output, *RMT* resting motor threshold (% of MSO), *AMT* active motor threshold (% of MSO), *SICI* short interval cortical inhibition, *ICF* intracortical facilitation, *CSP* cortical silent period

Superscript notes adjusted *p*-value significance following Holm multiple comparisons correction as follows—

^a^*p* > 0.05 (non-significant following correction)

^b^*p* > 0.05 (significant following correction)

**Fig. 1:**
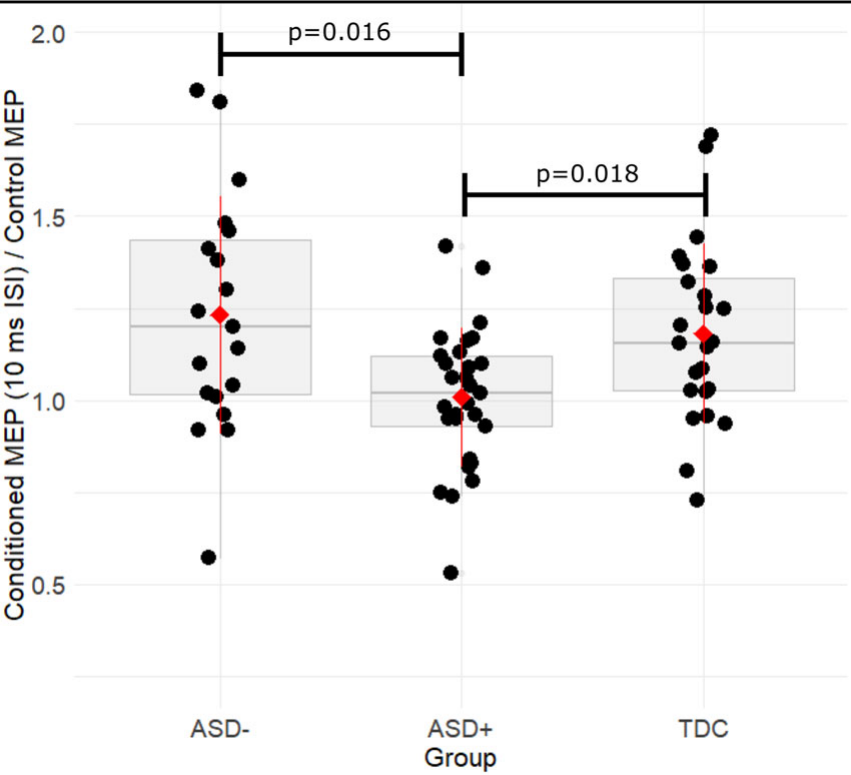
Boxplot visualization that ICF is significantly reduced in ASD+ compared to ASD− and TDC. Bracket lines indicate significant post-hoc comparisons. Larger ratios of ICF indicate greater paired pulse facilitation. Circles, individual subject average ICF; red diamond, overall group average; whiskers, group standard deviation; boxplot notch, group median; boxplot edges represent upper and lower quartiles. ASD autism spectrum disorder, ASD− without ADHD co-occurrence, ASD+ with ADHD co-occurrence, TDC typically developing control, ICF intracortical facilitation, MEP motor evoked potential

**Fig. 2:**
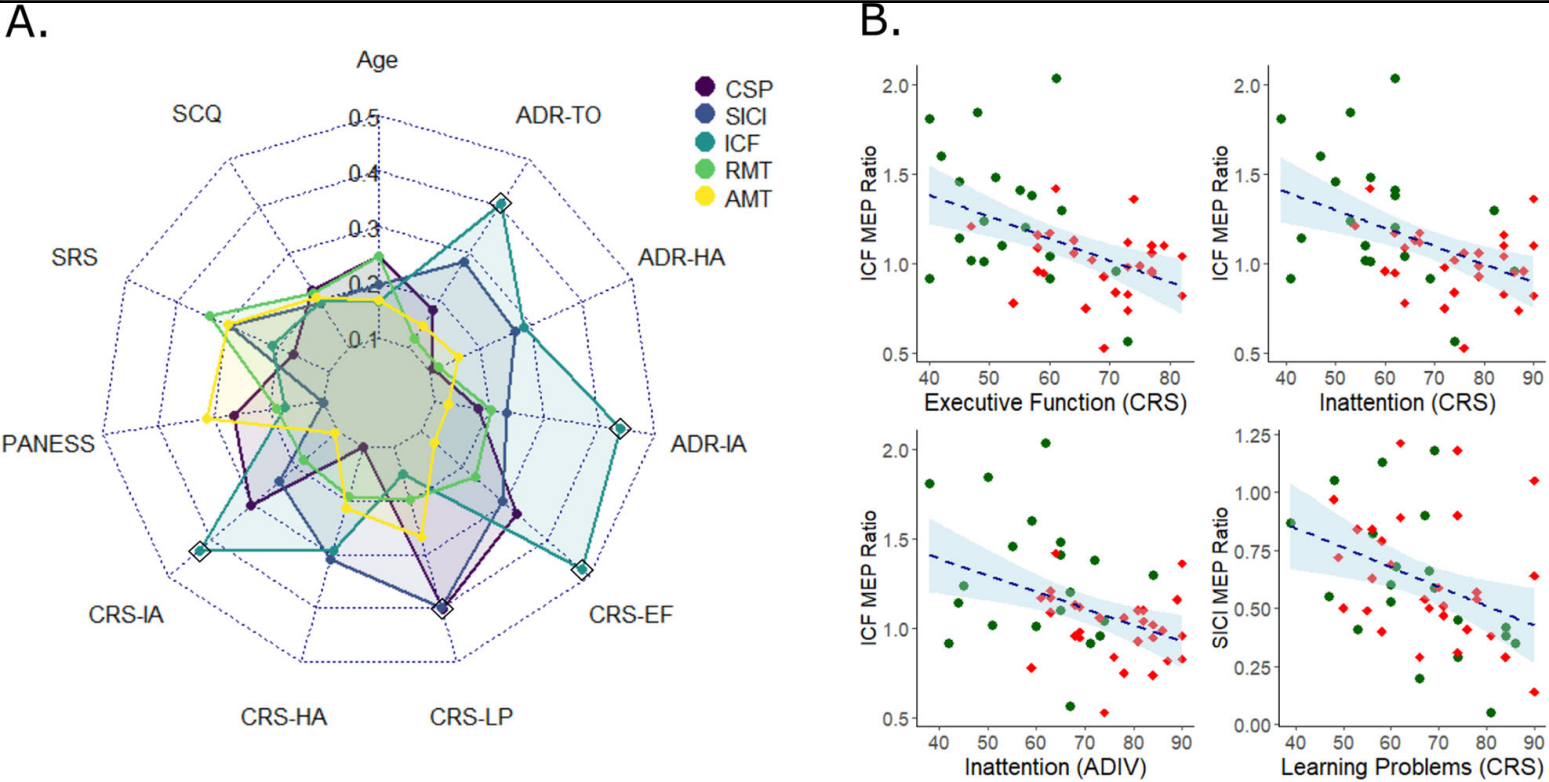
Clinical correlates of Resting Motor Physiology. **a** Radar plots of Spearman correlation coefficients between resting motor physiology measures (ICF, SICI, CSP, RMT) and various clinical measures. Absolute value of the correlation coefficient was plotted for comparison. Black outlined diamonds represent significant correlations. **b** Scatter plots of significant relationships (following FDR correction) between subject symptom severity and TMS measures. Green circles = ASD−, red diamond = ASD+. Higher clinical scores indicate more severe ADHD symptoms. ASD autism spectrum disorder, ASD− without ADHD co-occurrence, ASD+ with ADHD co-occurrence, CS*p* cortical silent period (ms), SICI short interval cortical inhibition: larger ratios indicate less inhibition, ICF intracortical facilitation: larger ratios indicate greater facilitation, RMT resting motor threshold (% of stimulator maximum); SCQ social communication questionnaire, SRS social responsiveness scale, PANESS physical and neurological examination for soft signs (raw scores; higher scores indicate more significant delays), EF executive function subscale, CRS Conners 3rd Edition; ADR ADHD rating scale IV

ICF was significantly diminished in ASD+ children compared to both ASD− and TDC youth (Fig. 1). Effect of stimulant medications: ASD+ subjects that where chronically taking stimulants (*n* = 17) and temporarily discontinued for the study and subjects untreated with stimulants (*n* = 12) did not have significantly different ICF (*p* = 0.33). Treated (*p* < 0.039) and untreated (*p* = 0.007) ASD+ groups both had significantly decreased ICF compared to the TD group. Correlations with resting motor physiology are presented in Table 3 and Table S1. Illustration of key demographic and clinical measures relationships with resting motor physiology is presented in Fig. 2.

**Table 3:** Pearson correlation (r) matrix for continuous variables across all ASD subjects

	CSP	SICI	ICF	RMT	AMT
*Motor physiology*					
CSP	1				
SICI	−0.427^a^	1			
ICF	−0.166	0.153	1		
RMT	0.301	−0.488^b^	0.079	1	
AMT	0.490^b^	−0.517 ^b^	−0.009	0.892^b^	1
*Measure*					
Age at visit date	0.184	0.121	0.083	−0.183	−0.085
Full-scale IQ	−0.289	0.184	−0.070	−0.254	−0.205
SCQ-total	0.151	−0.117	−0.121	0.146	0.134
SRS total raw score	0.088	−0.245	0.139	0.294	0.249
Total PANESS	0.206	−0.003	−0.09	0.107	0.265
*Behavioral ratings*					
ABC-irritability	0.178	−0.325	−0.104	0.252	0.256
ABC-social withdrawal	0.056	−0.044	0.040	0.121	0.096
ABC-stereotypy	−0.102	−0.143	0.016	0.215	0.111
ABC-hyperactivity	−0.037	−0.139	−0.172	0.122	0.124
ABC-inappropriate speech	−0.163	−0.058	0.065	0.184	0.099
CRS3 inattention	0.256	−0.171	−0.410^a^	−0.101	0.005
CRS3 hyperactivity	0	−0.260	−0.243	0.115	0.141
CRS3 learning problems	0.378^a^	−0.375^a^	−0.062	0.122	0.208
CRS3 executive functioning	0.285	−0.240	−0.477^b^	−0.160	−0.039
ADHDRSIV inattentive %-ile	0.100	-0.166	−0.422^a^	−0.129	−0.033
ADHDRSIV hyperactivity %-ile	−0.009	−0.210	−0.234	0.020	0.071
ADHDRSIV total %-ile	0.100	−0.229	−0.383^a^	−0.023	0.056

*CSP* cortical silent period (ms), *SICI* short interval cortical inhibition: larger ratios indicate less inhibition, *ICF* intracortical facilitation: larger ratios indicate greater facilitation, *RMT* resting motor threshold (% of stimulator maximum), *AMT* active motor threshold (% of stimulator maximum), *FSIQ* full-scale IQ, *SCQ* social communication questionnaire, *SRS* social responsiveness scale, *PANESS* physical and neurological examination for soft signs (raw scores; higher scores indicate more significant delays), *ABC* aberrant behavioral checklist, *CRS3* Conners 3rd Edition, *ADHDRS4* ADHD rating scale IV

^a^False discovery rate (FDR) adjusted *p*-value < 0.05

^b^FDR adjusted *p*-value < 0.01

## Discussion

### Introduction

Despite the high prevalence and clinical significance of ADHD co-occurrence in ASD, little is known regarding the underlying neural mechanisms that engender behavioral differences between ASD+ and ASD−. Intracortical interneurons within the motor cortex receive afferent inputs from other brain regions and play a critical role in modulating motor activity. MEP amplitude as a function of single and paired pulse TMS has been used as a surrogate marker for physiological changes associated with certain cognitive processors or disease states. Our primary interest was examining the clinical and behavioral correlates of motor cortex excitability differences, if any, across a large cohort of youth stratified by ADHD co-occurrence. We report a novel and robust finding, that ICF, a faciliatory TMS evoked paradigm, is reduced in ASD+ compared to ASD- and control subjects. Further, a decrease in ICF was related to worsening clinical and task-based measures of inattention and executive function.

### Rationale

Prior work by our group has established a robust relationship between reduced SICI, but not ICF, with increased ADHD severity and aberrant motor development across several cohorts of clinical populations including ADHD and Tourette’s syndrome with ADHD^12,34–36^. SICI has been associated with γ-aminobutyric acid (GABA)_A_ mediated inhibition at the level of the cortex^37^ and is associated with cortical interneuron activity. This finding supports a network model in which ADHD features are the product of dysfunctional GABAergic inhibition preventing the refinement of signals entering the motor cortex^38–40^ Thus, considering overlapping clinical presentations between ASD+ and ADHD, our expectation was to identify similar SICI changes associated with ADHD symptoms. Thus, we were surprised to see a marked reduction in ICF, without SICI differences, within the ASD+ group with clinically relevant associations.

### Relevance of ICF in ADHD

ICF is a TMS paradigm in which a subthreshold (60% of RMT) conditioning stimulus is paired with a suprathreshold (120%) test stimulus with an interstimulus interval of 10–15 milliseconds. This typically results in a higher amplitude (facilitation) than the test pulse alone^29^. Single-dose pharmacology studies have associated the ICF phenomena to excitatory, NMDAR-mediated neurotransmission in the cortex^37^. Most, but not all, previous studies in ADHD have shown no difference in ICF^41^. In addition to impaired SICI, Buchmann et al. also found an impaired ICF (ISI = 13 ms versus 10 ms in the present study) in an ADHD cohort of children compared to controls^42^. They further demonstrated a restoration of ICF following MPH administration. Similar studies in adults with ADHD are limited by sample sizes and conflict with existing pediatric studies. One out of two studies found a decrease in SICI and neither study found any difference in ICF^43,44^.Thus, in the present sample, the ASD+ finding of an impaired ICF with an intact SICI represents a distinct profile among studied neuropsychiatric disorders in children^41^.

### Physiology of ICF

Though previously ICF has been characterized as an exclusively cortical phenomena, recent reports have provided compelling evidence that ICF results from a complex interaction of cortical and subcortical circuits^45^. In contrast, SICI appears to be more strictly a phenomena isolated to the cortex^46^. Thus, the variability in ICF, even within similar individuals or disease -states, may additionally reflect dynamics related to deeper brain structures. Indeed, our ASD− and ASD+ cohort were finely phenotypically matched in terms of cognition, social functioning, and language abilities but differed widely in terms of inattention and hyperactivity symptoms. In youth with ADHD, a recent diffusion tensor imaging (DTI) study highlighted reduced grey matter concentration in the head of the left caudate as well as microstructural abnormalities in white matter density in the left middle temporal gyrus, right internal capsule, corpus callosum, and right midbrain^47^. Di Martino et al. explored functional neuroimaging correlates of ASD− and ASD+ youth by studying resting state fMRI local and global connectivity^48^. Diagnosis of ASD (regardless of the presence or absence of ADHD co-occurrence) was associated with abnormalities in the temporal lobe and amygdala. On the other hand, in ASD or TDC subjects with high levels of ADHD symptoms they identified increased local connectivity, associated with developmental immaturity, exclusively in subcortical structures including the right caudate, pallidum, and putamen. This local connectivity measure was also associated with CPRS total scores. The centrality of basal ganglia circuitry in ADHD has robust support in the literature and along with our observations may have a conserved underlying mechanism across disorders in which ADHD symptoms are present. Taken together, these findings suggest that ICF may reflect the aggregate effects of several aberrant subcortical neural circuits specifically relevant to ADHD symptoms in the ASD population.

The marked difference in ICF between ASD+ and ASD− may have special relevance for interpretation of [1 H] MRS studies of ASD. Spectroscopy studies of neurotransmitter levels in ASD remain nuanced and conflicting at times^49^. Thus far, there is emerging evidence that ICF is positively associated with [1 H] MRS measures of glutamate^50,51^. Dyke et al. studied the association of ICF with Glu/tCr levels in a 7 T [1 H] MRS. ICF demonstrated “substantial evidence” for a positive linear relationship with Glu/Cr levels using Bayesian classification (and a trend level Pearson’s correlation following Bonferroni correction (r = 0.52, *p* = 0.08^51^). No other paired pulse TMS measure in the study resulted in significant (or near significant) associations. Thus, our findings may partially explain a potential confounding factor in MRS studies which ASD is characterized without consideration for ADHD co-occurrence. For example, a recent meta-analysis of [1 H] MRS activity found that across studies, none of which accounted for ADHD co-occurrence in ASD, glutamate and Glx level varied widely based on age group or cortical region with no consistent behavioral correlates^52^.

### Conciliation with previous findings and SICI

Previous TMS investigations in heterogeneous samples of ASD subjects have not elicited differences in SICI or ICF^53,54^. Differences have emerged when ASD subjects are stratified by subgroups (thus limiting power) such as by early language delay^55,56^. The current study represents one of the largest samples of ASD youth to undergo TMS characterization (*n* = 59) and supports previous findings of essentially normal SICI. Thus, we opted to identify, if any exists, clinical characteristics that correlated with SICI and CSP values. Increased SICI (higher inhibition) and lengthened CSP, were highly correlated with the CPRS Learning Problems score reflecting academic struggles in reading, spelling, and/or math^57^. Since SICI was in the normal range, it is hard to interpret these findings without further study, including characterization of task-based SICI^58^.

We (and others) have previously reported that SICI is a marker for ADHD diagnosis and severity in otherwise typically developing youth^12,13^. The present findings suggest that based on TMS measures, there is a disassociation between ASD+ and ADHD such that, on average, ASD+ display intact SICI and elevated ICF and ADHD youth have reduced SICI and inconsistent ICF findings. This distinction may indicate that insufficient surround inhibition provided by GABAergic interneurons associated with SICI may be more central to attention and hyperactivity symptoms in ADHD, but other brain connectivity dysfunctions may be more prominently involved in ASD+.

### Relationships with clinical variables

We also report on several significant disease-relevant clinical and behavioral correlations with TMS measures. Diminished ICF is associated with worsening CPRS Inattention, CPRS Executive Function, ADHDRS-IV Inattentive Percentile, and ADHDRS-IV Total Percentile scores. The direction of this effect is internally consistent with the significant group difference between ASD− and ASD+. ICF did not correlate with subscales of hyperactivity or measures of social function. Though weakly correlated, lower ICF was related to worse attention and flexibility reaction times on computerized testing. However, these findings did not survive FDR correction and should be interpreted with caution. We speculate that one explanation, which would encourage further study into these preliminary findings, may relate to the nature of inattention in ASD versus ADHD. Despite widely different clinical severity in ADHD symptoms, both ASD− and ASD+ performed similarly on our computerized continuous performance battery.

In ASD, attention may represent difficulty in being able to flexibility shift attentional focus to non-preferred activities versus a the struggle to focus on the same matter over time as seen in ADHD^59^. Thus, performance on traditional CPT testing may reflect attentional domains more reflective of ADHD than inattention that may be due to special interests or social functioning in ASD.

### Limitations

Significant limitations to this study include the subjective nature of parent rating scales and the inherent heterogeneity within the ASD sample. However, the TMS measures are objective and quantitative and were performed blinded to the diagnostic group to minimize bias. As our primary focus was to identify the distinct resting motor physiology in ASD attributable to ADHD co-occurrence, our study cohort was limited to ASD subjects. To date, many groups (including our group) have reported paired-pulse TMS measures in ADHD and control subjects sufficient for meta-analysis^41^. Thus, we opted to apply identical methods, equipment, and stimulation parameters to maximize the extent our current results can be comparable to previously published results. Subject selection remains a challenge in pediatric TMS subjects. Higher resting thresholds in younger subjects made traditional paired pulse TMS measures using currently available stimulators impossible. As we have previously explored theta burst stimulation in the pediatric population, we are investigating methods of studying cortical excitability using subthreshold stimulation intensities, which may expand the inclusion pool to younger subjects. Finally, the inclusion of medication patients will likely have an effect across measurements including neuroleptics and SSRIs affecting ICF^37^. However, with current estimates demonstrating polypharmacy among autistic youth, a med free population would severely limited recruitment (and introduce other forms of selection biases)^60^.

### Future directions

The treatment implications of this finding will need further consideration. Of relevance, however, methylphenidate (MPH) administration, a highly efficacious treatment for ADHD, is frequently associated with increased ICF in ADHD^42^ and in controlled trials in healthy subjects^61–63^. Recent studies have implicated impaired glutamate signaling in some cases of ADHD and may represent an emerging treatment target. Thus, future study of acute MPH challenge may further clarify the relationship of TMS measures to treatment response. In addition, the current work is isolated to the motor cortex, but it is possible that specific abnormalities may exist upstream in other brain regions. Future studies may consider additional modalities including joint EEG/TMS to better understand other cortical contributions to the current findings.

## Conclusion

ADHD remains one of the most common and distressing co-occurring diagnoses in ASD with significant impact on prognosis. In the present study, we report a reduction of ICF in ASD+ from ASD− and TDC (and intact SICI). Along with recent discoveries regarding the physiology of ICF and association glutamatergic and subcortical circuits suggest that ADHD co-occurrence in ASD may have a distinct electrophysiological “signature”. ICF may constitute an emerging biomarker to study the physiology of ADHD in ASD, which may align with disease prognosis or treatment response.

## Supplementary information

Supplementary Table
