# Supplementary material for: Motor cortex facilitation: a marker of attention deficit hyperactivity disorder co-occurrence in autism spectrum disorder
Source: Transl Psychiatry. 2019 Nov 13;9:298. doi: 10.1038/s41398-019-0614-3 (PMC6853984; doi:10.1038/s41398-019-0614-3)
Supplement: Supplementary file 1 — Supplementary Table [file 41398_2019_614_MOESM1_ESM.docx]

Supplementary Materials

| **KiTap Domain** | **r** | **n** | **p** | **adj. p** |
| --- | --- | --- | --- | --- |
| Alertness RT (Mean) | 0.253 | 49 | 0.08 | 0.26 |
| Alertness RT (Median) | 0.289 | 49 | 0.04^a^ | 0.19 |
| Alertness RT (SD) | 0.114 | 49 | 0.43 | 0.60 |
| Distractibility RT (Mean) | 0.162 | 48 | 0.27 | 0.51 |
| Distractibility RT (Median) | 0.233 | 48 | 0.11 | 0.29 |
| Distractibility RT (SD) | -0.131 | 48 | 0.38 | 0.59 |
| Flexibility RT (Mean) | 0.340 | 46 | 0.02^a^ | 0.14 |
| Flexibility RT (Median) | 0.337 | 46 | 0.02^a^ | 0.14 |
| Flexibility RT (SD) | 0.200 | 46 | 0.18 | 0.40 |
| Go-No Go RT (Mean) | 0.085 | 47 | 0.57 | 0.68 |
| Go-No Go RT (Median) | 0.027 | 47 | 0.86 | 0.93 |
| Go-No Go RT (SD) | -0.111 | 47 | 0.46 | 0.60 |

Table S1: Spearman rank correlation (r) coefficient matrix for KiTap computerized testing and intracortical facilitation (ICF) across all ASD subjects (with and without ADHD co-occurrence). ASD, Autism Spectrum Disorder; RT, reaction time; n, number of subjects completing measure; adj. p, false discovery rate (FDR) adjusted p values; ^a^, p < 0.05 prior to FDR correction.
